# RETRACTION: Influence of Incentive Nursing Intervention on Recovery of Burn Patients after Vacuum Sealing Drainage

**DOI:** 10.1111/iwj.70649

**Published:** 2025-04-23

## Abstract

**RETRACTION:** RenL. , ZhangC. , ZhaoL. , LiC. , ZhangL. , and XueX. , “Influence of Incentive Nursing Intervention on Recovery of Burn Patients after Vacuum Sealing Drainage,” International Wound Journal 18, no. 6 (2021): 787–795, 10.1111/iwj.13582.33738955 PMC8613383

The above article, published online on 18 March 2021, in Wiley Online Library (http://onlinelibrary.wiley.com/), has been retracted by agreement between the journal Editor in Chief, Professor Keith Harding; and John Wiley & Sons Ltd. Following an investigation by the publisher, all parties have concluded that this article was accepted solely on the basis of a compromised peer review process. The editors have therefore decided to retract the article. The authors did not respond to our notice regarding the retraction.

**RETRACTION:** L. Ren , C. Zhang , L. Zhao , C. Li , L. Zhang , and X. Xue , “Influence of Incentive Nursing Intervention on Recovery of Burn Patients after Vacuum Sealing Drainage,” International Wound Journal 18, no. 6 (2021): 787–795, 10.1111/iwj.13582.33738955 PMC8613383

The above article, published online on 18 March 2021, in Wiley Online Library (http://onlinelibrary.wiley.com/), has been retracted by agreement between the journal Editor in Chief, Professor Keith Harding; and John Wiley & Sons Ltd. Following an investigation by the publisher, all parties have concluded that this article was accepted solely on the basis of a compromised peer review process. The editors have therefore decided to retract the article. The authors did not respond to our notice regarding the retraction.

